# Pattern Recognition Receptors and Cytokines in *Mycobacterium tuberculosis* Infection—The Double-Edged Sword?

**DOI:** 10.1155/2013/179174

**Published:** 2013-11-12

**Authors:** Md. Murad Hossain, Mohd-Nor Norazmi

**Affiliations:** ^1^School of Health Sciences, Universiti Sains Malaysia, 16150 Kubang Kerian, Kelantan, Malaysia; ^2^Institute for Research in Molecular Medicine, Universiti Sains Malaysia, 16150 Kubang Kerian, Kelantan, Malaysia

## Abstract

Tuberculosis, an infectious disease caused by *Mycobacterium tuberculosis* (Mtb), remains a major cause of human death worldwide. Innate immunity provides host defense against Mtb. Phagocytosis, characterized by recognition of Mtb by macrophages and dendritic cells (DCs), is the first step of the innate immune defense mechanism. The recognition of Mtb is mediated by pattern recognition receptors (PRRs), expressed on innate immune cells, including toll-like receptors (TLRs), complement receptors, nucleotide oligomerization domain like receptors, dendritic cell-specific intercellular adhesion molecule grabbing nonintegrin (DC-SIGN), mannose receptors, CD14 receptors, scavenger receptors, and FC*γ* receptors. Interaction of mycobacterial ligands with PRRs leads macrophages and DCs to secrete selected cytokines, which in turn induce interferon-*γ*- (IFN*γ*-) dominated immunity. IFN*γ* and other cytokines like tumor necrosis factor-*α* (TNF*α*) regulate mycobacterial growth, granuloma formation, and initiation of the adaptive immune response to Mtb and finally provide protection to the host. However, Mtb can evade destruction by antimicrobial defense mechanisms of the innate immune system as some components of the system may promote survival of the bacteria in these cells and facilitate pathogenesis. Thus, although innate immunity components generally play a protective role against Mtb, they may also facilitate Mtb survival. The involvement of selected PRRs and cytokines on these seemingly contradictory roles is discussed.

## 1. Introduction

Despite 100 years of research, infection with *Mycobacterium tuberculosis *(Mtb) is still a major health problem worldwide and one of the main causes of human death by a particular infectious agent. Every year, 10 million new cases are diagnosed, and 2 million people die because of tuberculosis (TB) [1]. The incidence of TB is increasing in association with increased numbers of HIV/AIDS patients. The problem was further worsened by the emergence of multidrug resistant (MDR) and extensively drug resistant (XDR) Mtb [2]. Host immunity is usually capable of inhibiting Mtb growth in most individuals and often leads to latent infection, which is characterized by the confinement of live but inactive bacteria in the granuloma. According to the World Health Organization (WHO), one-third of the world's population is infected with latent TB, but only 10% of it develops active TB [3]. The possibility of developing active TB is increased with the incidence of immune-compromising conditions such as AIDS and diabetes. This indicates that the host immune system is active in most individuals and provides protection against Mtb infection. The host defense mechanisms against mycobacterial infection are largely divided into two phases: innate immunity and adaptive immunity. This review focuses on the natural, innate immunity against Mtb, although the two arms of the host immune response complement each other in defense against Mtb.

TB begins with the engulfment of Mtb through inhalation into the pulmonary alveoli. After inhalation, the first line of host defense starts with the recognition of Mtb by phagocytic cells of the innate immune system such as macrophages and dendritic cells (DCs). Macrophages and DCs express numerous pattern recognition receptors (PRRs) that recognize antigenic molecules expressed on Mtb called pathogen-associated molecular patterns (PAMPs) [4–6]. Among the various PRRs, toll-like receptors (TLRs) seem to play the most important role in Mtb infection. These receptors interact with pathogen-specific ligands, facilitate uptake of Mtb, and initiate an intracellular signaling cascade in host cells that induces production of cytokines [7] which are essential to elicit the adaptive immune response and arrest bacterial growth. Thus, TLRs serve as a link between innate and adaptive immune defense against infection with mycobacteria. The proinflammatory cytokines produced through mycobacterial recognition of PRRs in turn regulate antimycobacterial mechanisms of macrophages such as generation of reactive nitrogen intermediates (RNI) and reactive oxygen intermediates (ROI) [8]. Interferon-*γ* (IFN*γ*), in combination with tumor necrosis factor-*α* (TNF*α*), upregulates nitric oxide synthase 2 (NOS2) expression and induces production of RNI within the phagolysosome, thus mediating intracellular Mtb killing [9]. Anti-inflammatory cytokines, however, usually facilitate Mtb infection by opposing proinflammatory immune responses of host cells [9]. An extensive knowledge about host immune responses is essential for understanding the pathophysiology of TB.

In the last several years, immunologists have made substantial progress in our understanding of host innate immune response to Mtb and on the specific roles of different immune components and effectors in the process of host protection. However, mycobacteria possess the ability to evade destruction by the innate host defense mechanisms [10]. Thus understanding the nature of the innate response is crucial for us to be able to modulate it to prevent Mtb infection. This review focuses on our current understanding of the recognition of Mtb by the PRRs and subsequent regulation of host innate immune response to Mtb specifically on the cellular response to cytokines secreted as well as their role in protection of the host or promotion of Mtb pathogenesis. More extensive reviews on the subject can also be obtained from previous studies (see, e.g., [2, 3, 6, 8, 10–16]). 

## 2. Pathogenesis of *M. tuberculosis*

Mtb usually infects the lungs where the tubercle bacilli are inhaled into the pulmonary alveoli. Here, we discuss the pathogenesis of Mtb mainly on the basis of studies that have been performed in animal models, particularly in mice. After inhalation, the alveolar resident macrophages initially ingest the bacilli. Most bacilli are destroyed at this stage. Mtb that evades the initial destruction will multiply which then results in disruption of the macrophages and may manifest as active TB disease [11, 12]. In most cases however, chemokines are released by the alveolar macrophages, which lead to the recruitment of monocyte-derived DCs and macrophages to the lungs [11, 12]. These newly recruited cells readily ingest but usually do not destroy the bacilli. At this stage, Mtb grows logarithmically with nominal tissue damage [11, 12]. At the same time, blood monocytes accumulate at the infection site. T-cell mediated immunity then develops after two to three weeks of infection [11, 12, 16]. Antigen-specific T lymphocytes that migrate to the infection site will multiply within the early lesions or tubercles and release proinflammatory cytokines such as IFN*γ* which in turn activates macrophages to kill the intracellular mycobacteria [17]. Eventually, bacillary growth stops, and the central dense necrosis in primary lesions (granuloma) contains the extracellular growth of mycobacteria. This phase of infection is known as the latent phase where the growth of mycobacteria is put in check. Reactivation of latent TB can occur months or years afterwards when the immune system is weakened [9, 18, 19].

## 3. Is Innate Immunity Adequate for Host Protection?

**Figure 1:**
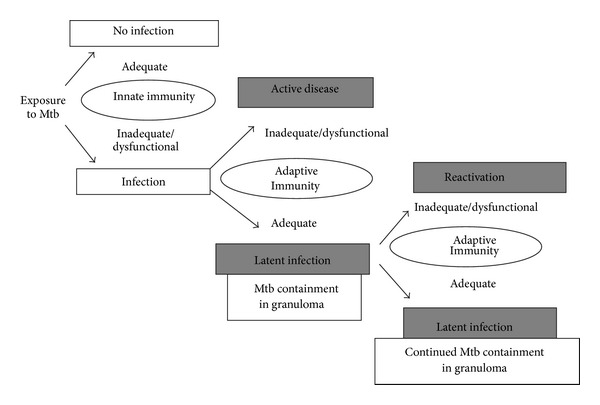
Pathogenesis and transmission profile of Mtb. Adequate innate immunity is believed to protect the host from Mtb infection in the majority of people, while inadequate innate immune response leads to Mtb infection. The adaptive immunity then restricts Mtb growth which leads to Mtb containment in granulomas which causes latent TB infection. Defective adaptive immunity causes active TB and promotes reactivation of the latent TB.

After inhalation, alveolar macrophages engulf the Mtb bacilli and initiate the innate immune response as a first line of host defense. Innate and adaptive immunity are synergistic, and innate immune responses orchestrate the initiation of downstream adaptive immune responses.  Figure 1 illustrates the transmission profile of TB in the Mtb-infected host. Upon exposure to Mtb, a major percentage of individuals remain uninfected probably because of the expression of adequate innate immunity. However, we hypothesize that an inadequate innate immunity in some individuals is unable to contain the infection and adaptive immune responses are thus needed to arrest growth of bacteria and to confer host protection. The adaptive immunity provides protection against the development of active TB, and these individuals remain latently infected. Latent infections are susceptible to be reactivated when the hosts' immunity weakens or when they become immunecompromised [9, 18, 19]. In the worst case scenario, a defective adaptive immune response in individuals with inadequate innate immunity leads to early progression of active TB. Thus a high level of host innate immune response itself can effectively protect Mtb infection, while a defective or impaired innate immune response requires the help of adaptive immune response to furnish protection against Mtb infection. 

This notion however is rather simplistic since it does not take into consideration the initial bacterial load, the environmental influence, and the host's immunogenetics. These three factors can modulate the effectiveness of the innate immune response. A low-dose mycobacterial load induces a Th1 type immune response while a high-dose challenge with mycobacteria is known to shift cellular responses towards Th2 type response [20, 21]. Mice immunized with low-dose mycobacteria induced Th1 response and showed partial protection against Mtb H37Rv challenge, while mice immunized with high-dose mycobacteria primed a mixed Th1/Th2 response, predominantly Th2 response, that overcomes the initial protection provided by Th1 response and showed massive pneumonia [20, 21]. The nature of immune response is also influenced by environmental factors such as the presence of environmental mycobacteria which prime Th1 type responses, whereas infection with soil transmitted helminths induces Th2 type responses and stimulates T-regulatory cells which secrete anti-inflammatory cytokines such as interleukin-10 (IL-10) and transforming growth factor-*β* (TGF-*β*) [22, 23]. Host's genetic factors also influence the nature of immune response and could thereby determine the fate of Mtb infection on whether it will manifest as a clinical disease [24]. Genetic factors are shown to contribute to TB incidences, with an estimated heritability ranging from 36 to 80% [25]. Moreover, diverse lineages and different strains of Mtb have been shown to induce highly heterogeneous immune responses upon infection, and the incidence of TB relies upon their ability to alter innate immune responses and the secretion of cytokines by host phagocytic cells [26–30]. Thus there appears to be a complex interaction between the host, pathogen, and the environment which determines the type and efficacy of the immunity induced by the Mtb infection to progress to active disease or to be contained as latent infection or eliminated by the host.

## 4. Innate Immune Recognition of *M. tuberculosis*

Phagocytosis is the key mechanism of defense against Mtb infection after it is being inhaled. The alveolar resident phagocytic cells, macrophages and DCs, engulf Mtb bacilli and cause initiation of infection. The PAMPs of Mtb are recognized by specific PRRs, expressed on phagocytic cells, and allow entry of the bacilli into the cells which is central in initiation and coordination of the host innate [31] and subsequently adaptive immune response. This PRR-dependent entry is also important as a key determinant of the fate of Mtb. Although phagocytosis is mainly involved in the killing of pathogen, the growth of some Mtb bacilli can also be promoted depending on the route of entry into phagocytic cells. Prior to phagocytosis, Mtb or Mtb components are recognized by a variety of different PRRs that include TLRs, complement receptors (CRs), scavenger receptors, nucleotide-binding-oligomerization-domain- (NOD-) like receptors (NLRs), dectin-1, surfactant protein A (Sp-A) receptors, mannose receptors (MRs), and the dendritic cell-specific intercellular adhesion molecule grabbing nonintegrin (DC-SIGN) [32–34].

### 4.1. Immune Recognition of *M. tuberculosis*: Role of Toll-Like Receptors in Phagocytosis and *M. tuberculosis* Killing

**Figure 2:**
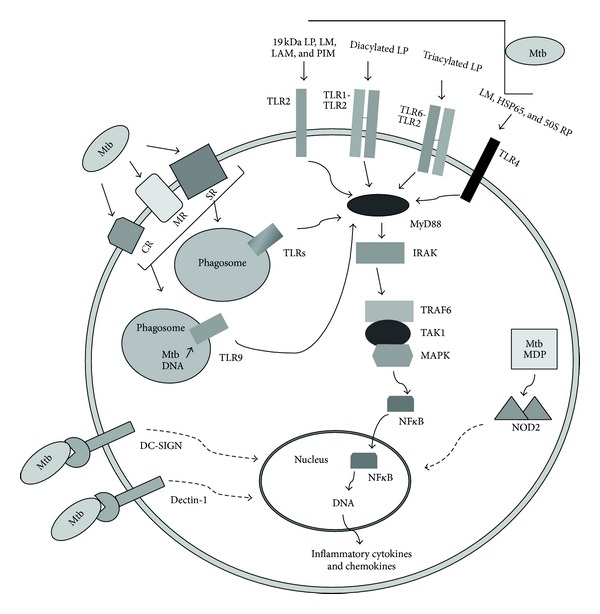
Immune recognition of *M. tuberculosis* during phagocytosis. Several pattern recognition receptors (PRRs) on phagocytic cells have been identified for the recognition of conserved pathogen associated molecular patterns (PAMPs) of mycobacteria. TLR2 recognizes the 19 kDa lipoprotein (LP), lipomannan (LM), and lipoarabinomannan (LAM). TLR1-TLR2 and TLR6-TLR2 heterodimers bind diacylated and triacylated LP, respectively. TLR4 binds tri- and tetra-acylated LM, heat shock protein 65 (HSP65), and 50S ribosomal protein (50S RP), whereas mycobacterial DNA is recognized by phagosomal TLR9. Complement receptors (CRs) are mainly responsible for uptake of opsonized *M. tuberculosis*, while mannose receptors (MRs) and scavenger receptors (SR) are for uptake of nonopsonized *M. tuberculosis*. Cytosolic receptor NOD2 interacts with Mtb derived peptidoglycan component muramyl dipeptide (Mtb-MDP). MyD88 is the key component in TLR-mediated defense mechanism whose downstream signaling cascade leads to the activation of NF-*κ*B transcription factor and to the production of inflammatory molecules. TLRs are expressed on phagocytic cell surface and in phagosomes. Dashed arrow represents the presence of numerous signaling molecules in between.

TLRs are phylogenetically conserved receptors expressed, among other cells, on macrophages and DCs which recognize PAMPs of pathogens [35]. Interaction of specific mycobacterial ligands with TLRs leads to macrophage activation which triggers signaling pathways (Figure 2) in which the adaptor molecule, myeloid differentiation primary response protein 88 (MyD88), plays a central role [36]. MyD88 links mycobacterial ligand bound TLRs to the interleukin-1 receptor-associated kinase (IRAK) which subsequently recruits TNF receptor-associated factor 6 (TRAF6), TGF-*β*-activated protein kinase 1 (TAK1), and mitogen-activated protein kinase (MAPK) in a signaling pathway that mediates translocation of the nuclear transcription factor NF-*κ*B into the nucleus. NF-*κ*B promotes transcription of the genes of host innate immune defense and subsequent expression of proinflammatory cytokines and chemokines (Figure 2) [37–41]. IL-12 and IL-18 induce IFN*γ*-dominated immunity, which involves natural killer (NK), Th1, and CD8+ T cells, activates macrophages, and leads to bacterial growth inhibition. Activation of macrophages initiates phagosome maturation and promotes antimycobacterial effector mechanisms such as autophagy, interferon inducible GTPase such as Irgm-1/LRG47 and induces production of nitric oxide and other RNIs [8, 12, 15, 42]. 

Among the TLRs identified, TLR2, TLR4, and TLR9 are the key receptors that are involved in recognition of Mtb. Mtb expresses various antigenic ligands that interact with TLR2 and subsequently activate MyD88 mediated signaling pathway. Among these, LpqH, a 19-kDa secreted lipoprotein of Mtb, interacts with TLR2 and induces production of TNF*α* and nitric oxide from both murine and human macrophages [43]. LpqH also induces IL-12 production from human monocytes [43]. TLR2 is shown to recognize other mycobacterial lipoproteins such as LprA (Rv1270) [44] and LprG (Rv1411c) [45]. TLR2 also interacts with lipomannan [46] and phosphatidyl-*myo*-inositol mannoside (PIM) [47, 48] to induce immune response [48]. TLR2 forms heterodimers with either TLR1 or TLR6. The heterodimers TLR2-TLR1 and TLR2-TLR6 interact with diacylated and triacylated lipoproteins, respectively [48, 49]. TLR2^−/−^ mice show defects in formation of granuloma [50]. When challenged with high dose Mtb, TLR2^−/−^ mice show greater susceptibility to infection than wild type mice [50, 51]. Furthermore, TLR2^−/−^ mice show defect in protection of chronic infection with Mtb [50]. TLR2-mediated activation of macrophages enhanced the expression of genes of vitamin D receptor and vitamin-D-1-hydroxylase which in turn promotes the production of the antimicrobial peptide, cathelicidin, and provides resistance to Mtb [52]. TLR2 is essential for production of TNF*α*, as inhibitory TLR2 (TLR2-P681H) expression in mouse RAW TT10 macrophage cells absolutely inhibits TNF*α* production induced by whole Mtb [36]. Consistent with the deduced role of TLR2 in studies using TLR2 knockout mice/macrophages, host genetics that causes even a single mutation in TLR2 gene has been shown to significantly affect the incidence of TB. For example, individuals with TLR2 Arg753Gln single nucleotide polymorphisms (SNPs) are found to be more susceptible to TB compared to healthy controls of a Turkish cohort [53]. Several other SNPs such as TLR2 Arg677Trp, TLR2 597CC, and TLR2 T597C also show an association with susceptibility to TB in different cohorts [54–56] indicating that the TLR2 polymorphisms influence the susceptibility to Mtb infection.

Like TLR2, TLR4 also induces MyD88-mediated signaling pathway upon recognition of selected PAMPs and regulates the production of proinflammatory cytokines [51] (Figure 2). In addition to MyD88 pathway, stimulation of TLR4 also activates MyD88 independent TIR-domain containing adapter-inducing interferon-*β* (TRIF) pathway where TRIF links to interferon regulatory factor-3 (IRF3) which induces interferon-*β* secretion, and this cytokine subsequently activates a series of genes that regulate host protection [37, 57]. A recent study showed that certain strains of Mtb preferentially activate TLR4, whereas others stimulate TLR2 and induce distinct immune responses depending on the TLR that recognizes Mtb [30]. Mtb heat shock protein 65 (HSP65) was shown to signal exclusively through TLR4 as macrophages from TLR4^−/−^ mice showed no response to Mtb HSP65, whereas Mtb HSP70 was shown to signal through both TLR4 and TLR2 [58]. Another *in vitro* study showed that Mtb chaperonin 60.1 (Cpn60.1) protein predominantly stimulates TLR4 to induce subsequent immune signaling, whereas Mtb Cpn60.2 can use both TLR2 and TLR4 for its signaling for cytokine production [59]. Recombinant Mtb 50S ribosomal protein Rv0652 stimulates macrophages in a TLR4-dependent and MyD88-dependent signalling pathway to induce secretion of proinflammatory cytokines, predominantly TNF and chemokine such as monocyte chemoattractant protein 1 [60]. The 38-kDa glycolipoprotein of Mtb H37Rv signals through both TLR2 and TLR4 and activates MAPKs, which then induces expression of TNF*α* and IL-6 in human monocytes during mycobacterial infection [61]. TLR4 antagonist E5531 substantially blocked Mtb induced TNF*α* production in RAW 264.7 macrophages and primary human alveolar macrophages [62]. Although several molecules are shown to activate TLR4 *in vitro*, the actual ligands that TLR4 recognizes during mycobacterial infection still remain unknown. Moreover, *in vivo* study showed that TNF*α* production was greatly reduced in TLR4-deficient mice [63]. TLR4 defective mice (C3H/HeJ) showed greater susceptibility to Mtb infection in comparison to wild type mice [63], but these mice have been shown to display greater resistance than wild type models upon exposure to low dose Mtb [51]. Further studies are thus needed to elucidate the role of TLR4 in Mtb infection. 

The role of TLR9 in Mtb infection has recently been revealed. TLR9 interacting with mycobacterial DNA activates macrophages and induces proinflammatory cytokine synthesis [64]. TLR9 in combination with TLR2 was studied to detect both their combined and individual roles [65]. TLR9^−/−^ mice infected with Mtb exhibited defective production of IL-12p40 and IFN*γ*. However, in the presence of TLR2, TLR9^−/−^ mice showed some extent of resistance to low dose Mtb aerosol challenge indicating that TLR2 may compensate for TLR9 deficiency [65]. Expectedly TLR2/9 double knock-out mice showed extensive susceptibility to Mtb infection. The DCs and macrophages of mice deficient either in TLR2 or TLR9 also showed significantly greater defects in response to IL-12. TLR9 in combination with TLR2 seems to cooperate with each other in defence against Mtb infection [65].

While the role of TLRs in Mtb recognition and subsequent induction of immune responses were mostly performed in mouse models and in macrophages, very little is known on their role in DC maturation and cytokine production by DCs. TLRs mediated interaction (phagocytosis) of Mtb causes activation and maturation of DCs which then transport the pathogen to draining lymph nodes and present Mtb antigens to naïve T cells, thus initiating adaptive immune responses to Mtb [10]. DCs induce production of numerous cytokines including TNF*α*, IL-6, IL-10, and IL-12 upon infection with Mtb *in vitro* [66, 67]. Another study showed that secretion of IL-6 and IL-10 by DCs largely depends on TLR2-mediated recognition of Mtb and the production of IL-6 and IL-10 was significantly reduced in the absence of TLR2 signaling in TLR2^−/−^ DCs [67]. Both TLR2 and TLR4 on human DCs were found to be activated by muramyl dipeptide derivatives with a single octanoyl or stearoyl fatty acid chain [68]. 

### 4.2. Non-TLR Receptor-Mediated Phagocytosis of *M. tuberculosis*

Phagocytosis of Mtb can occur via a variety of different receptors other than TLRs, expressed on the phagocytic cellular surface (Figure 2). Host cellular response may be influenced by the type of receptor utilized for internalization of Mtb. Thus, there is no specific route of Mtb entry into phagocytic cells. However, the fate of Mtb survival can be influenced by the route of entry as some of these receptors favor host protection, while some others promote mycobacterial infection.

*Complement Receptors.* Complement receptors (CRs) are membrane proteins expressed on the surface of phagocytic cells. They interact specifically with complement components, some of which are essential for opsonization of mycobacteria prior to phagocytosis. Complement receptors, namely, CR1, CR3, and CR4, on the phagocytic cell surface are involved in promoting Mtb ingestion [69]. In fact Mtb utilizes such cell surface molecules to gain access into macrophages. Complement component C3 binds to the surface of Mtb and enhances phagocytosis by macrophages through binding with CR1, CR3, and CR4 [69–71]. CR3 is relatively more important among the CRs. In the absence of CR3, human monocytes and macrophages showed about 70 to 80% reduced ability in phagocytosis of Mtb [70, 71]. CRs can mediate uptake of virulent as well as avirulent strains of Mtb, and blocking of CRs inhibits the phagocytosis of Mtb [70]. Furthermore, CR3-Mtb interaction can prevent formation of respiratory bursts which causes phagosomal arrest of early endosomes and produces no inflammatory response [72]. The entry of Mtb through binding with CRs thus appears as a beneficial route for Mtb survival and pathogenesis. 

*Mannose Receptors.* Mannose receptors (MRs) are transmembrane C-type (calcium dependent) lectins that bind certain sugars, specifically mannose, present on the surface of pathogens. Both monocyte-derived and tissue macrophages express MRs that are mainly associated with Mtb phagocytosis without prior opsonization. MRs interact with the terminal mannose residues of lipoarabinomannan (LAM) expressed on the cell surface of virulent strains of Mtb (Erdman strain) [73]. Interaction of Mtb derived mannosylated lipoarabinomannan (ManLAM) with MR promotes inhibition or delay in phagosome-lysosome fusion [74]. Thus MRs mediate entry of Mtb into macrophages and facilitate the survival and expansion of Mtb due to inhibition of phagosome-lysosome fusion [74, 75]. While CRs recognize both avirulent and virulent strains of Mtb, MRs are reported to recognize only virulent strains [70], indicating that this path of entry is particularly useful for pathogenesis of virulent Mtb.

*NOD-Like Receptors.* The NOD (nucleotide oligomerization domain) like receptors (NLRs) are a class of cytoplasmic protein containing a series of leucine-rich repeats in the C-terminal region, similar to other PRRs of the innate immune system, which interact with the PAMPs of the pathogen. NOD2, a member of NLRs, interacts with bacterial peptidoglycan component muramyl dipeptide (MDP). Mice deficient in NOD2 showed defective cytokine production upon infection with Mtb [76]. Mutation of the *NOD2* gene has been reported to cause defects in cytokine production by mononuclear cells against Mtb [77]. Live but not heat-killed Mtb activates the NOD2-mediated signaling pathway [78]. Live Mtb, though localized in the phagosomal compartment within macrophages, induces phagosomal membrane damage, results in Mtb interaction with cytosolic NOD2 receptor, and consequently stimulates the NOD2 pathway [79], which indicates that NLRs-mediated signaling pathway provides protection to host against Mtb infection.

*CD14 Receptors.* CD14 receptors mainly recognize LPS and play a role in phagocytosis. In fetal microglia, blocking of CD14 receptors by anti-CD14 antibodies causes inhibition of Mtb phagocytosis [80]. Phagocytic cells expressing high levels of CD14 such as porcine alveolar macrophages preferentially ingest *M. bovis*, and anti-CD14 antibodies inhibit bacillary entry [81]. However, one study [80] suggested that CD14 is not essential in phagocytosis, but its expression is upregulated as a consequence of Mtb infection, which in turn may promote the pathogen's capability to modulate the immune response. Thus, the role of CD14 in Mtb internalization by macrophages is ambiguous.

*Dectin-1.* Dectin-1 is a PRR which consists of an extracellular carbohydrate recognition domain with an intracellular tyrosine activated motif. Macrophages, DCs, neutrophils, and a subset of T-cells are the main cells that express dectin-1 receptor. *β*-Glucans present in fungal pathogens are the ligands of dectin-1. However, this receptor can also recognize *α*-glucan expressed on the surface of some species of Mtb [82]. Dectin-1-mediated recognition of Mtb induces Th1 and Th17 responses, and subsequent secretion of proinflammatory cytokines that promote bacterial killing [83]. Dectin-1 also promotes IL12p40 production by DCs in response to Mtb infection, and blocking of dectin-1-dependent activity or DCs from dectin-1^−/−^ mice results in reduced production of Mtb induced IL12p40 production [84]. Although dectin-1 is suggested to play a protective role in Mtb infection [84, 85], Marakalala et al. [86] showed that dectin-1, that signals through the Syk/CARD9 pathway, may augment host susceptibility to Mtb as pulmonary bacilli burdens were observed lower in dectin-1-deficient mice. Moreover, CARD9-deficient mice were shown to be highly susceptible to Mtb and died early after high dose Mtb H37Rv aerosol challenge [87]. Dectin-1 is essential for production of TNF*α*, RANTES, IL-6, and G-CSF by murine macrophages upon mycobacterial infection [85, 88]. Further studies are necessary to reveal the exact role of this receptor in immunity against mycobacterial infection, in particular, to reveal the mycobacterial ligand(s) recognised by this receptor as mycobacteria do not possess *β*-glucans. 

*Scavenger and Fc γ* *Receptors.* Scavenger receptors (SRs) and Fc*γ* receptors (Fc*γ*R) play a less important role in Mtb pathogenesis. When uptake by CRs is being inhibited, Mtb may enter into phagocytic cells through binding with type-A scavenger receptors [89]. Fc*γ*R mediates uptake of IgG-opsonized mycobacteria, induces generation of ROIs, and promotes formation of phagolysosome, thus aiding in Mtb killing [90]. 

*DC-SIGN.* Dendritic cell-specific intercellular adhesion molecule grabbing nonintegrin (DC-SIGN) is a carbohydrate recognition receptor expressed mainly on DCs. This receptor serves as PRR as well as adhesion receptor, because of its association with migration of DCs and interaction between DCs and T-cells [91, 92]. DC-SIGN recognizes Man-LAM and lipomannans expressed on the cell surface of mycobacteria and induces IL-10 production, thereby promoting an anti-inflammatory response by DCs [93]. DC-SIGN on DCs interacts with Man-LAM to internalize *M. bovis* BCG, and blocking of DC-SIGN by using anti-DC-SIGN antibody restricts the infection of DCs [93]. However, the DC-SIGN homologue, SIGNR3 in a murine model, was shown to confer early resistance against Mtb infection, and the resistance was impaired in SIGNR3-deficient mice. During infection, SIGNR3 is expressed in lung phagocytes and interacts with Mtb bacilli and mycobacterial surface glycoconjugates and mediates host protection by inducing secretion of protective cytokines including TNF*α* and IL-6 [94, 95]. It was shown in an *in silico* study that two variants (−871G and −336A) in the promoter region of DC-SIGN gene (CD209) may confer protection against TB [96]. However, genetic studies on human population showed that sequence and length variation in DC-SIGN have no significant impact on TB susceptibility [97, 98]. 

*Surfactant Protein A.* Surfactant protein A (SP-A) is a soluble receptor protein present in abundance in host alveolar macrophages. SP-A induces an increased phagocytosis of Mtb by human macrophages [99]. HIV-positive patients with Mtb infection showed increased expression of SP-A in their lungs and resulted in a threefold higher risk of developing active disease [100]. SP-A mediated uptake of Mtb by human macrophages significantly reduces the generation of RNI in the macrophages and thereby decreases macrophage cytotoxicity and aids in intracellular survival of Mtb [99, 101]. 

Thus Mtb employs various mechanisms involving PRRs to get access into alveolar macrophages. The host cellular responses depend on the receptor used which determines the fate of Mtb infection whether the bacteria will survive or be destroyed by the initial inflammatory responses. Hence whether Mtb will be eliminated/contained or escape the host innate immunity may be dependent on the sum total of PRR interactions with the bacteria. Therefore, further studies are needed to assess whether some or all of these mechanisms are operative under *in vivo* conditions in order to gain a better understanding on the development of the disease. 

## 5. *M. tuberculosis *Induced Cytokine Secretion: Protection for Host or for Pathogen?

Interaction of Mtb ligands with the PRRs on macrophages and DCs causes cell activation and secretion of cytokines which regulates the innate immune response as well as initiates the adaptive immune response to Mtb. Cytokines, produced in response to Mtb infection, are not always necessarily favorable to host protection, and some of these immune modulators favor the pathogen. Studies showed that cytokines, in response to a particular pathogen, produce beneficial as well as deleterious effects to the host. In this section, we discuss the importance of cytokines induced during Mtb infection under two major groups, proinflammatory cytokines (cytokines that are generally known to be beneficial to the host) and anti-inflammatory cytokines (cytokines that are generally deleterious to the host), with some cytokines displaying both pro- and anti-inflammatory properties. 

### 5.1. Proinflammatory Cytokines

Th1 immunity plays a vital role for protection against TB. In response to Mtb infection, immune cells produce several proinflammatory cytokines such as TNF*α*, IFN*γ*, IL-1*β*, IL-6, IL-12, IL-18, and IL-23, which are involved in killing of the mycobacteria.

*Tumor Necrosis Factor- α* *(TNF α*). TNF*α* is a prototype proinflammatory cytokine which is produced by phagocytic cells activated with mycobacteria or mycobacterial components and plays a key role in host defense against Mtb infection [102]. TNF*α* is crucial in containment of latent infection in mice granuloma [103] and protects the host from developing active TB. TNF*α* production is also detected at the site of infection in TB patients [104]. Mice deficient in either TNF*α* production or TNF*α* receptor exhibit increased susceptibility to Mtb infection [105, 106]. Mice treated with anti-TNF*α* antibody or lacking TNF*α* receptor are more vulnerable to infection with mycobacteria [107, 108]. TNF*α* secretion by Mtb-infected macrophages stimulates production of RNIs in conjunction with IFN*γ* [109] and promotes apoptosis [110] and thereby induces killing of Mtb. 

TNF*α* is the main cytokine that promotes granuloma formation which contains the infectious foci and thus prevents dissemination [103, 111]. Neutralization of TNF*α* activity during the latent phase of TB induces reactivation of the disease in C57BL/6 mice and causes severe tissue damage and death [103]. The most significant findings of this study were the apparent loss of granuloma structure and the infiltration of cells throughout the lungs which led to a destructive immunopathology [103]. Clay et al. [112] showed that loss of TNF signaling enhances mortality in *M. marinum *infected zebra fish. The authors also showed that intracellular bacterial growth and granuloma formation were increased in the absence of TNF, which were followed by necrotic death of macrophages and granuloma breakdown. These findings suggest that TNF is not required for granuloma formation but for maintaining the integrity of granuloma structure by limiting bacterial growth inside macrophages and for preventing their necrosis [112]. Treatment with anti-TNF*α* has also been shown to boost the reactivation of latent TB in patients with Crohn's disease and rheumatoid arthritis [113]. 

*Interferon- γ* *(IFN γ*). IFN*γ* is a key cytokine that mediates antigen-specific T-cell immunity in response to Mtb infection. *In vitro* expression of mycobacterial antigen-specific IFN*γ* can be a potential substitute marker of Mtb infection [114]. Various immune cells including CD4+ and CD8+ T cells, B cells, NK cells, NKT cells, and antigen presenting cells such as monocytes, macrophages, and DCs produce IFN*γ*. IFN*γ* promotes antigen presentation, recruits CD4+ T-lymphocytes and/or cytotoxic T lymphocytes (CTL), and thereby mediates mycobacterial killing. An *in vitro* study shows that IFN*γ* is capable of inhibiting Mtb growth in mouse but not in human macrophages [115]. GKO mice lacking IFN*γ* show greatest susceptibility to Mtb infection [116]. Exposure to Mtb induces severe infections to individuals defective in IFN*γ* or IFN*γ* receptor gene [117]. However, Mtb is shown to depress IFN*γ* production in patients with active disease [118]. Mtb is also shown to inhibit macrophages from responding adequately to IFN*γ* [119]. 

Production of IFN*γ* is mostly regulated by two other cytokines such as IL-12 and IL-18 which mediate IFN*γ* production upon infection with Mtb in cellular innate immune response [120, 121]. Upon recognition of mycobacterial ligands, macrophages secrete IL-12 and chemokines (e.g., macrophage-inflammatory protein-1*α* (MIP-1*α*)). These chemokines recruit NK cells at the infection site where IL-12 and IL-18 promote IFN*γ* production which then exerts protective immune response [122, 123]. 

*Interleukin-12 (IL-12).* IL-12 is a proinflammatory cytokine that plays a key role in host defense against Mtb infection. Phagocytic cells produced IL-12 upon phagocytosis of Mtb [124]. IL-12 together with IL-18 induces IFN*γ* production and drives Th1 response that is essential for host defense against Mtb infection. IL-12-deficient mice are at high risk of Mtb infection. IL-12 supplementation to the Mtb susceptible Balb/c mice leads to killing of bacilli at the initial stage of the disease [125]. IL-12 expression is detected in lung infiltrates, pleurisy, and granulomas of patients with active TB [126, 127]. The expression of IL-12 receptors is also increased at the site of infection [128]. IL-12 receptor beta 1 deficiency has also been recognized in a patient with abdominal TB [129]. Deleterious mutations in IL-12 and IL-12 receptor genes have been reported in patients suffering from nontuberculous mycobacterial infections [130, 131]. These patients lacking effective IL-12 and IL-12 receptor show a reduced capacity of IFN*γ* production. In fact, IL-12 exerts its protective roles against mycobacterial infection mainly through induction of IFN*γ*, thus serving as a link between innate and adaptive host immune responses [132]. 

*Interleukin-18 (IL-18).* IL-18 is a proinflammatory cytokine, which in synergy with IL-12 induces IFN*γ* production. This cytokine can also induce production of other proinflammatory cytokines, chemokines, and transcription factors [133]. Thus, IL-18 plays a protective role against mycobacterial infections. IL-18 knockout mice show greater susceptibility to BCG and Mtb [134]. Higher expression of IL-18 correlates with resistance in mice against *M. leprae *infection [135]. In fact, both IL-18 and IFN*γ* were expressed in TB pleurisy [136]. 

*Interleukin-1 β* *(IL-1 β*). IL-1*β* is a proinflammatory cytokine which is mainly produced by monocytes, macrophages, and DCs [11] and is involved in host immune response to Mtb. IL-1*β* is expressed at the site of infection in TB patients and is important in Mtb control [137]. Mice unable to respond to IL-1*α* and IL-1*β* display enhanced susceptibility to Mtb infection and induce defective granuloma formation [138, 139]. IL-1 was shown to mediate signals through IL-1 receptor (IL-1R) in response to mycobacterial infection [140]. Mice deficient in MyD88, the common adaptor molecule of TLR/IL-1R receptor family, were shown to be highly susceptible to Mtb infection, suggesting the importance of signaling through this pathway in innate defense against the bacterium [141, 142]. Although the importance of TLR/TLR-ligand interactions is well evidenced in immune response to Mtb, controversy remains on whether MyD88 mediated protection requires the signaling from TLRs or via cytokine signaling through IL-1R [3, 41, 143]. To examine the particular role of TLR versus IL-1R mediated signals in MyD88 dependent defense against Mtb, Mayer-Barber et al. [144] used Mtb infected MyD88^−/−^, TRIF/MyD88^−/−^, IL-1R1^−/−^, and IL-1*β* ^−/−^ mice and observed that all four groups showed highly increased pulmonary bacterial burden with acute mortality. These observations suggest that MyD88 regulates host protection to Mtb through IL-1*β*/IL-1R1 signaling rather than TLR-mediated signaling of cytokine production. These findings were further supported by the fact that the absence of IL-1R signal causes impairment in early control of Mtb infection similar to that seen in the absence of MyD88 [145]. The authors also concluded that IL-1/IL-1R mediated signaling is an important element of MyD88 dependent immune response and that IL-1/IL-1R mediated signal might be involved for the most part of MyD88-dependent host response to regulate acute Mtb infection. 

*Interleukin-6 (IL-6).* IL-6 possesses both pro- and anti-inflammatory properties and is produced by phagocytic cells at the onset of mycobacterial infection [137]. IL-6 plays multiple and sometimes seemingly opposing roles in the immune system, including inflammation, hematopoiesis, and differentiation of T cells. IL-6-deficient mice showed enhanced susceptibility during early infection with Mtb where T-cell mediated adaptive immunity is yet to fully develop [146]. On the contrary, IL-6 inhibits the production of protective cytokines such as TNF*α* and IL-1*β* and facilitates *M. avium *growth *in vitro* [147]. 

*Interleukin-23 (IL-23).* IL-23 is a proinflammatory cytokine and is secreted by activated monocytes, macrophages, and DCs [148]. IL-23 together with IL-12 promotes activation of Ag-specific CD4+ T cells in the draining lymph nodes of Mtb-infected lungs [149]. Mice lacking in both IL-12 and IL-23 are markedly susceptible to infection with Mtb. However, mice deficient in IL-12 alone exhibit a partial compromise of protective immunity to Mtb, which suggests a protective role for IL-23 [150]. In contrast, mice deficient only in IL-23 showed effective control of Mtb infection, suggesting that IL-23 is not crucial for host protection [151]. IL-23 was shown not to be necessary for the early control of Mtb growth; however, it was implicated as an essential element for the control of Mtb growth late in infection as mice deficient in the p19 component of IL-23 (Il23a^−/−^) exhibited increased bacterial growth at this stage [152]. IL-23 was also required for the development and maintenance of Th17 responses to Mtb infection [152]. Plasmids encoding both chains of IL-23 and IL-12 were reported to increase the protection induced by a DNA vaccine (namely, DNA85B) against Mtb aerosol challenge [153]. Another study showed that exogenous IL-23 is required for the development of IFN*γ* secreting Th1 cells but not for the development of protective Th17 cells in IL12p40^−/−^ mice [154]. However, this group in a recent study showed that in the absence of IL-12 and IL-23, both Th17 and Th1 BCG-specific T cells neither were expanded nor provided protection against Mtb [155]. A reduced Th17 response was observed to be directly correlated with the clinical outcome of Mtb infection, and the frequency of Th17 cells was lower in active TB patients than that of healthy individuals as well as individuals infected with latent TB [156]. Suppression of Th17 responses by inhibiting Th17 cell activation was also found to be associated with the incidence of active TB [157]. Therefore, further studies are needed to justify the role of IL-23 in the development of Th17 responses against Mtb infection. 

### 5.2. Anti-Inflammatory Cytokines

Anti-inflammatory cytokines are the immunoregulatory molecules that inhibit cell activation and the induction of proinflammatory cytokines and thus counter the inflammatory process. These cytokines act mainly by inhibiting the production of proinflammatory cytokines or by opposing their biological effects. By the same token, overexpression of anti-inflammatory cytokines or dysregulation of their expression may compromise protection afforded by the proinflammatory cytokines. IL-4, IL-10, and TGF-*β* are the major anti-inflammatory cytokines secreted by immune cells.

*Interleukin-4 (IL-4).* IL-4 is an anti-inflammatory cytokine which causes deleterious effects in TB causing deactivation of macrophage and suppression of IFN*γ* production [158]. IL-4 in association with IL-13 can impair macrophage function by inducing alternative activation which causes downregulation of TLR2 and TNF and increases soluble TNF receptors, DC-SIGN, and IL-10 production [159–163]. IL-4 can be considered as a potential marker for active TB as it is expressed in high levels in TB patients especially in the tropics [164–166]. Overexpression of IL-4 induces progressive disease [167] and reactivation of latent infection [168] in mice infected with Mtb. Increased production of IL-4 has also been detected in TB patients, especially those with cavitary disease [169]. 

*Interleukin-10 (IL-10).* IL-10 is an anti-inflammatory cytokine which is produced by macrophages and T cells upon infection with Mtb. IL-10 deactivates macrophage function [170] by downregulating IL-12 and TNF*α* expression, which in turn reduces production of IFN*γ* by T cells and thus aids in Mtb survival. IL-10 inhibits CD4+ T cell responses and opposes antigen presentation by cells infected with mycobacteria [171]. IL-10 impairs macrophage function in transgenic mice infected with mycobacteria and causes them to be susceptible to the infection [172]. Although CD8+ T cells are generally thought to provide protection against Mtb infection, IL-10 secreting phenotype of CD8+ T cells may occur during chronic infection which causes the cells to display a dysfunctional and immunosuppressive response [173]. IL-10 inhibits phagosome maturation, which in turn facilitates Mtb survival and disease outgrowth [174]. IL-10 also inhibits IFN*γ* mediated activation of macrophages which leads to decreased secretion of ROIs and RNIs and thus impairs intracellular Mtb killing mechanisms [175–177]. Moreover, IL-10 was also shown to limit antigen presentation by downregulating major histocompatibility complex molecules [177]. DCs process mycobacterial antigens to carry them to draining lymph nodes in an IL-12p40 dependent mechanism, which was shown to be retarded by IL-10 [178]. DCs mediate T-cell differentiation and promote recruitment of Th1 cells to the lungs of Mtb infected mice which were suggested to be restricted by the inhibitory effect of IL-10 on the production of the chemokine CXCL10 [179]. Neutralization of endogenous IL-10 in peripheral blood mononuclear cells (PBMC) of pulmonary TB patients enhanced T-cell proliferation and IFN*γ* production [180]. Elevated levels of IL-10 and TGF-*β* were found in bronchoalveolar lavage fluid and comprised mainly macrophages and neutrophils, in pulmonary TB patients [181]. Increased levels of IL-10 and Mtb antigen CFP32 were observed in the sputum of pulmonary TB patients suggesting a positive correlation between IL-10 and the progression of disease [182]. Overexpression of IL-10 by CD4+ T-cells in transgenic mice causes them to be more susceptible to *M. bovis* BCG and Mtb [172, 183]. Blocking of IL-10R signaling with anti-IL-10R monoclonal antibody during chronic infection boosts T-cell recruitment in the infection site and increases T-cell mediated IFN*γ* production, thereby providing enhanced protection with reduced bacterial loads [184]. Treatment of Mtb infected IL-10^−/−^ mice with anti-IL-10R monoclonal antibody leads to reduce bacterial burdens compared to the control counterparts [179]. Moreover, IL-10^−/−^ mice were also shown to have increased level of protection against nontubular mycobacterial infections, such as *M. avium* and BCG [185, 186]. However, the role of IL-10 in TB remains controversial. IL-10^−/−^ mice were shown to have similar bacterial loads in the lung and enhanced levels of IFN*γ* production during early infection compared to wild type control mice, though the elevated IFN*γ* failed to provide any additional protection [187, 188]. On the light of the above discussion, it can be concluded that IL-10 downregulates host Th1 immune responses and thereby favors mycobacterial infection. 

*Transforming Growth Factor- β* *(TGF- β* *).* Like IL-4 and IL-10, TGF-*β* is also an anti-inflammatory cytokine that counteracts protective immunity in TB. Upon stimulation with Mtb ligands, human monocytes and DCs produce TGF-*β* which is found in the granulomatous lesions of TB patients [189, 190]. TGF-*β* exerts various anti-inflammatory effects that include inhibition of macrophage-mediated production of ROI and RNI [191], suppression of T cell proliferation [192], interference with NK and cytotoxic T lymphocyte function, and downregulation of proinflammatory cytokine release [193]. Addition of TGF-*β* in coculture of mononuclear phagocytes and Mtb causes inhibition of phagocytosis and facilitates Mtb growth in a dose-dependent manner [189]. Inhibitors of TGF-*β* abolish the anti-inflammatory effects of the cytokine and thereby protect the host from Mtb infection [194]. 

Thus like PRRs, the balance of various cytokine production may influence the outcome of infection and disease progression.

### 5.3. Type I Interferons: Friend or Foe? 

Type I IFNs are a group of cytokines that mainly include multiple forms of IFN*α* and one IFN*β*, which mediate signals through a common receptor (IFN-*α*/*β* receptor (*Ifnar*)). Type I IFNs are generally associated with innate immune response to viral infection. However, the innate immune response of type I IFNs can also be induced by bacterial infections [195]. 

The role of type I IFNs in TB is not completely understood. In contrast to the well-known role of type II IFN (IFN*γ*) in the immune control of TB, type I IFNs are reported to promote rather than to control Mtb infection. Monocytes of patients with active but not latent TB produce type I IFN. Furthermore, transcriptional activities of leukocytes induced by type I IFNs were observed in active but not latent infection. These findings are suggestive of type I IFN to be involved directly in causing active TB [196, 197]. TPL2-ERK1/2 signaling pathway mediates host protection against Mtb infection through negative regulation of type I IFN production [198]. An increased level of type I IFNs was produced in the absence of TPL2 which promoted IL-10 production and exacerbated disease [198]. However, a recent study showed that type I IFNs play a nonredundant protective role against TB. Mice deficient in both type I and type II IFN receptors (Ifnar^−/−^Ifngr^−/−^) showed more severe lung histopathology and died significantly earlier than did mice with impaired type II IFN signaling (Ifngr^−/−^) alone [195]. Type I IFNs showed beneficial effect against pulmonary TB [199] and have been suggested to be used as a potential therapy against infections with MDR strains [200] or in patients lacking IFN*γ* receptor [201]. However, IFN*α* treatment in a patient with viral hepatitis was found to upregulate the severity of TB [202]. Redford et al. [203] showed that type I IFN signaling leads to the enhancement of mycobacterial growth in mice coinfected with influenza A virus and Mtb.

Type I IFNs also display both useful and harmful effects during mycobacterial infections *in vitro*. Type I IFNs impair normal macrophage function and promote BCG growth in human macrophages [204], whereas IFN*β* inhibits growth of BCG by increasing human DC maturation [205]. Mtb-induced type I IFNs can selectively limit the production of IL-1*β* in human macrophages, thus promoting TB infection [206]. These controversies are probably due to the fact that type I IFNs act differently on distinct cell types [207]; hence more extensive *in vivo* investigation may need to be carried out to further delineate their function. Type I IFNs also show distinct effects in response to infection by different strains of mycobacteria. IFN-*β* increases the resistance of mice to *M. avium* infection [208], whereas mice infected with the virulent Mtb HN878 strain become vulnerable by intranasal treatment with IFN-*α*/*β* [209]. A recent study also showed that different strains of Mtb result in distinct profile of IFN-*β* production by bone marrow derived macrophages during infection [30]. Type I IFNs are able to control early infection with BCG in Ifnar^−/−^mice [210], but late infection with the Mtb HN878 strain [211]. This time-dependent relationship between type I IFNs and their overlapping biological activities requires further investigation. 

The role of type I IFNs as pro- or anti-inflammatory effectors is still uncertain. It is assumed that type I IFNs demonstrate anti-inflammatory effects when IFN*γ* is present in abundance during TB. Type I IFNs inhibit inflammasome activation and thereby inhibit inflammasome-mediated IL-1*β* production [212]. In addition, type I IFNs activate signal transducers and activators of transcription 3 (STAT3) which in turn inhibits STAT1-dependent gene activation and thereby downregulates the activities of immune inflammatory mediators in myeloid cells [213]. Type I IFNs-activated STAT3 also induces IL-10 production and intensifies anti-inflammatory responses [213]. Finally, the ubiquitous presence of both type I and type II IFN receptors implies that many cell types can respond to these cytokines but with distinct outcomes.

## 6. Concluding Remarks

We have gained a better understanding of innate immune mechanisms with the discovery of TLRs at the end of last century. TLRs are largely involved in mycobacterial uptake which initiates a signaling pathway to trigger production of innate immune effector molecules, cytokines, and chemokines. These molecules dictate the innate immune system, initiate the adaptive immune response to Mtb, and finally mediate Mtb growth arrest or killing. The non-TLRs-mediated or TLR independent mechanisms of Mtb phagocytosis have also been shown to induce protective innate immune response to mycobacteria and subsequently initiate adaptive immune defense. Both innate and adaptive immunity are synergistic in biological systems and exert protective response against invading pathogen. Therefore, both innate and adaptive immune responses are important and have pivotal roles in host defense against mycobacteria. However, there still remain some fundamental issues to be answered regarding the innate immune responses to Mtb. 

One such issue is how Mtb is able to evade innate immune defense mechanisms. Mtb can maintain intracellular growth inside the phagosome by inhibiting phagolysosome formation. Recognition of PRRs and the consequent innate immune response in killing Mtb is well reported, but the reasons why it fails to restrict Mtb growth and infection remain incompletely understood. Further studies are thus needed to elucidate why some people develop active TB upon infection while others do not.

Another vital concern in TB is the latent infection and its reactivation. It is believed that innate immunity is primarily needed to restrict Mtb growth in the initial phase of infection. After inhalation, alveolar resident macrophages engulf Mtb bacilli, where it faces a series of immunological events and the immune machinery finally provides protection to the host. However, this protection sometimes leads to latent infection by containment of mycobacteria within granulomas, which may reactivate in immunecompromised individuals. However, the role of innate immune systems in latency and in reactivation is still poorly understood. Therefore, a precise understanding of the innate immune response to Mtb infection is crucial to design novel vaccination strategies that can enhance preexisting immunity or can prevent reactivation of latent TB.

## References

[1] World Health Organization Global tuberculosis control.

[2] Ahmad S (2011). Pathogenesis, immunology, and diagnosis of latent mycobacterium tuberculosis infection. *Clinical and Developmental Immunology*.

[3] Korbel DS, Schneider BE, Schaible UE (2008). Innate immunity in tuberculosis: myths and truth. *Microbes and Infection*.

[4] Medzhitov R, Janeway CJ (2000). The Toll receptor family and microbial recognition. *Trends in Microbiology*.

[5] Kaisho T, Akira S (2000). Critical roles of Toll-like receptors in host defense. *Critical Reviews in Immunology*.

[6] Takeda K, Kaisho T, Akira S (2003). Toll-like receptors. *Annual Review of Immunology*.

[7] Aderem A, Ulevitch RJ (2000). Toll-like receptors in the induction of the innate immune response. *Nature*.

[8] Flynn JL, Chan J (2001). Immunology of tuberculosis. *Annual Review of Immunology*.

[9] Flynn JL, Chan J (2003). Immune evasion by Mycobacterium tuberculosis: living with the enemy. *Current Opinion in Immunology*.

[10] Bhatt K, Salgame P (2007). Host innate immune response to Mycobacterium tuberculosis. *Journal of Clinical Immunology*.

[11] Kleinnijenhuis J, Oosting M, Joosten LAB, Netea MG, van Crevel R (2011). Innate immune recognition of mycobacterium tuberculosis. *Clinical and Developmental Immunology*.

[12] van Crevel R, Ottenhoff THM, van der Meer JWM (2002). Innate immunity to Mycobacterium tuberculosis. *Clinical Microbiology Reviews*.

[13] Zuniga J, Torres-Garcia D, Santos-Mendoza T, Rodriguez-Reyna TS, Granados J, Yunis EJ (2012). Cellular and humoral mechanisms involved in the control of tuberculosis. *Clinical & Developmental Immunology*.

[14] Li Y, Wang Y, Liu X (2012). The role of airway epithelial cells in response to mycobacteria infection. *Clinical & Developmental Immunology*.

[15] Saiga H, Shimada Y, Takeda K (2011). Innate immune effectors in mycobacterial infection. *Clinical and Developmental Immunology*.

[16] Tsolaki AG (2009). Innate immune recognition in tuberculosis infection. *Advances in Experimental Medicine and Biology*.

[17] Wolf AJ, Desvignes L, Linas B (2008). Initiation of the adaptive immune response to Mycobacterium tuberculosis depends on antigen production in the local lymph node, not the lungs. *The Journal of Experimental Medicine*.

[18] Tufariello JM, Chan J, Flynn JL (2003). Latent tuberculosis: mechanisms of host and bacillus that contribute to persistent infection. *Lancet Infectious Diseases*.

[19] Flynn JL, Chan J (2005). What’s good for the host is good for the bug. *Trends in Microbiology*.

[20] Hernandez-Pando R, Pavön L, Arriaga K, Orozco H, Madrid-Marina V, Rook G (1997). Pathogenesis of tuberculosis in mice exposed to low and high doses of an environmental mycobacterial saprophyte before infection. *Infection and Immunity*.

[21] Power CA, Wei G, Bretscher PA (1998). Mycobacterial dose defines the Th1/Th2 nature of the immune response independently of whether immunization is administered by the intravenous, subcutaneous, or intradermal route. *Infection and Immunity*.

[22] Elias D, Britton S, Kassu A, Akuffo H (2007). Chronic helminth infections may negatively influence immunity against tuberculosis and other diseases of public health importance. *Expert Review of Anti-Infective Therapy*.

[23] Rook G, Nor N, Acosta A, Elena Sarmiento M (2010). Tuberculosis in developing countries due to high-dose challenge in partially immune individuals. *The Art and Science of Tuberculosis Vaccine Development*.

[24] Bellamy R (2003). Susceptibility to mycobacterial infections: the importance of host genetics. *Genes and Immunity*.

[25] Möller M, de Wit E, Hoal EG (2010). Past, present and future directions in human genetic susceptibility to tuberculosis. *FEMS Immunology and Medical Microbiology*.

[26] Portevin D, Gagneux S, Comas I, Young D (2011). Human macrophage responses to clinical isolates from the Mycobacterium tuberculosis complex discriminate between ancient and modern lineages. *PLoS Pathogens*.

[27] Wang C, Peyron P, Mestre O (2010). Innate immune response to mycobacterium tuberculosis Beijing and other genotypes. *PLoS One*.

[28] Krishnan N, Malaga W, Constant P (2011). Mycobacterium tuberculosis lineage influences innate immune response and virulence and is associated with distinct cell envelope lipid profiles. *PLoS One*.

[29] Sarkar R, Lenders L, Wilkinson KA, Wilkinson RJ, Nicol MP (2012). Modern lineages of Mycobacterium tuberculosis exhibit lineage-specific patterns of growth and cytokine induction in human monocyte-derived macrophages. *PloS One*.

[30] Carmona J, Cruz A, Moreira-Teixeira L, Sousa C (2013). Strains are differentially recognized by TLRs with an impact on the immune response. *PloS One*.

[31] Akira S, Uematsu S, Takeuchi O (2006). Pathogen recognition and innate immunity. *Cell*.

[32] Jo E-K (2008). Mycobacterial interaction with innate receptors: TLRs, C-type lectins, and NLRs. *Current Opinion in Infectious Diseases*.

[33] Harding CV, Boom WH (2010). Regulation of antigen presentation by Mycobacterium tuberculosis: a role for Toll-like receptors. *Nature Reviews Microbiology*.

[34] El-Etr SH, Cirillo JD (2001). Entry mechanisms of mycobacteria. *Frontiers in Bioscience*.

[35] Sandor F, Buc M (2005). Toll-like receptors. I. Structure, function and their ligands. *Folia Biologica*.

[36] Underhill DM, Ozinsky A, Smith KD, Aderem A (1999). Toll-like receptor-2 mediates mycobacteria-induced proinflammatory signaling in macrophages. *Proceedings of the National Academy of Sciences of the United States of America*.

[37] Takeda K, Akira S (2004). TLR signaling pathways. *Seminars in Immunology*.

[38] Akira S (2003). Toll-like receptor signaling. *Journal of Biological Chemistry*.

[39] Quesniaux V, Fremond C, Jacobs M (2004). Toll-like receptor pathways in the immune responses to mycobacteria. *Microbes and Infection*.

[40] Ryffel B, Fremond C, Jacobs M (2005). Innate immunity to mycobacterial infection in mice: critical role for toll-like receptors. *Tuberculosis*.

[41] Reiling N, Ehlers S, Hölscher C (2008). MyDths and un-TOLLed truths: sensor, instructive and effector immunity to tuberculosis. *Immunology Letters*.

[42] Bafica A, Feng CG, Santiago HC (2007). The IFN-inducible GTPase LRG47 (Irgm1) negatively regulates TLR4-triggered proinflammatory cytokine production and prevents endotoxemia. *Journal of Immunology*.

[43] Brightbill HD, Libraty DH, Krutzik SR (1999). Host defense mechanisms triggered by microbial lipoproteins through toll-like receptors. *Science*.

[44] Pecora ND, Gehring AJ, Canaday DH, Boom WH, Harding CV (2006). Mycobacterium tuberculosis LprA is a lipoprotein agonist of TLR2 that regulates innate immunity and APC function. *Journal of Immunology*.

[45] Gehring AJ, Dobos KM, Belisle JT, Harding CV, Boom WH (2004). Mycobacterium tuberculosis LprG (Rv1411c): a novel TLR-2 ligand that inhibits human macrophage class II MHC antigen processing. *Journal of Immunology*.

[46] Quesniaux VJ, Nicolle DM, Torres D (2004). Toll-like receptor 2 (TLR2)-dependent-positive and TLR2-independent-negative regulation of proinflammatory cytokines by mycobacterial lipomannans. *Journal of Immunology*.

[47] Gilleron M, Himoudi N, Adam O (1997). Mycobacterium smegmatis phosphoinositols-glyceroarabinomannans: structure and localization of alkali-labile and alkali-stable phosphoinositides. *Journal of Biological Chemistry*.

[48] Jones BW, Means TK, Heldwein KA (2001). Different Toll-like receptor agonists induce distinct macrophage responses. *Journal of Leukocyte Biology*.

[49] Thoma-Uszynski S, Stenger S, Takeuchi O (2001). Induction of direct antimicrobial activity through mammalian toll-like receptors. *Science*.

[50] Drennan MB, Nicolle D, Quesniaux VJF (2004). Toll-like receptor 2-deficient mice succumb to Mycobacterium tuberculosis infection. *American Journal of Pathology*.

[51] Reiling N, Hölscher C, Fehrenbach A (2002). Cutting edge: toll-like receptor (TLR)2- and TLR4-mediated pathogen recognition in resistance to airborne infection with Mycobacterium tuberculosis. *Journal of Immunology*.

[52] Liu PT, Stenger S, Li H (2006). Toll-like receptor triggering of a vitamin D-mediated human antimicrobial response. *Science*.

[53] Ogus AC, Yoldas B, Ozdemir T (2004). The Arg753Gln polymorphism of the human Toll-like receptor 2 gene in tuberculosis disease. *European Respiratory Journal*.

[54] Ben-Ali M, Barbouche M-R, Bousnina S, Chabbou A, Dellagi K (2004). Toll-like receptor 2 Arg677Trp polymorphism is associated with susceptibility to tuberculosis in Tunisian patients. *Clinical and Diagnostic Laboratory Immunology*.

[55] Caws M, Thwaites G, Dunstan S (2008). The influence of host and bacterial genotype on the development of disseminated disease with Mycobacterium tuberculosis. *PLoS Pathogens*.

[56] Thuong NTT, Hawn TR, Thwaites GE (2007). A polymorphism in human TLR2 is associated with increased susceptibility to tuberculous meningitis. *Genes and Immunity*.

[57] Akira S, Takeda K, Kaisho T (2001). Toll-like receptors: critical proteins linking innate and acquired immunity. *Nature Immunology*.

[58] Bulut Y, Michelsen KS, Hayrapetian L (2005). Mycobacterium tuberculosis heat shock proteins use diverse toll-like receptor pathways to activate pro-inflammatory signals. *Journal of Biological Chemistry*.

[59] Cehovin A, Coates ARM, Hu Y (2010). Comparison of the moonlighting actions of the two highly homologous chaperonin 60 proteins of Mycobacterium tuberculosis. *Infection and Immunity*.

[60] Kim K, Sohn H, Kim J-S (2012). Mycobacterium tuberculosis Rv0652 stimulates production of tumour necrosis factor and monocytes chemoattractant protein-1 in macrophages through the Toll-like receptor 4 pathway. *Immunology*.

[61] Jung S-B, Yang C-S, Lee J-S (2006). The mycobacterial 38-kilodalton glycolipoprotein antigen activates the mitogen-activated protein kinase pathway and release of proinflammatory cytokines through Toll-like receptors 2 and 4 in human monocytes. *Infection and Immunity*.

[62] Means TK, Jones BW, Schromm AB (2001). Differential effects of a Toll-like receptor antagonist on Mycobacterium tuberculosis-induced macrophage responses. *Journal of Immunology*.

[63] Branger J, Leemans JC, Florquin S, Weijer S, Speelman P, van der Poll T (2004). Toll-like receptor 4 plays a protective role in pulmonary tuberculosis in mice. *International Immunology*.

[64] Hemmi H, Takeuchi O, Kawai T (2000). A Toll-like receptor recognizes bacterial DNA. *Nature*.

[65] Bafica A, Scanga CA, Feng CG, Leifer C, Cheever A, Sher A (2005). TLR9 regulates Th1 responses and cooperates with TLR2 in mediating optimal resistance to Mycobacterium tuberculosis. *The Journal of Experimental Medicine*.

[66] Hickman SP, Chan J, Salgame P (2002). Mycobacterium tuberculosis induces differential cytokine production from dendritic cells and macrophages with divergent effects on naive T cell polarization. *Journal of Immunology*.

[67] Jang S, Uematsu S, Akira S, Salgame P (2004). IL-6 and IL-10 induction from dendritic cells in response to mycobacterium tuberculosis is predominantly dependent on TLR2-mediated recognition. *Journal of Immunology*.

[68] Uehori J, Fukase K, Akazawa T (2005). Dendritic cell maturation induced by muramyl dipeptide (MDP) derivatives: monoacylated MDP confers TLR2/TLR4 activation. *Journal of Immunology*.

[69] Ferguson JS, Weis JJ, Martin JL, Schlesinger LS (2004). Complement protein C3 binding to Mycobacterium tuberculosis is initiated by the classical pathway in human bronchoalveolar lavage fluid. *Infection and Immunity*.

[70] Schlesinger LS (1993). Macrophage phagocytosis of virulent but not attenuated strains of Mycobacterium tuberculosis is mediated by mannose receptors in addition to complement receptors. *Journal of Immunology*.

[71] Schlesinger LS, Bellinger-Kawahara CG, Payne NR, Horwitz MA (1990). Phagocytosis of Mycobacterium tuberculosis is mediated by human monocyte complement receptors and complement component C3. *Journal of Immunology*.

[72] Sturgill-Koszycki S, Haddix PL, Russell DG (1997). The interaction between Mycobacterium and the macrophage analyzed by two-dimensional polyacrylamide gel electrophoresis. *Electrophoresis*.

[73] Schlesinger LS, Kaufman TM, Iyer S, Hull SR, Marchiando LK (1996). Differences in mannose receptor-mediated uptake of lipoarabinomannan from virulent and attenuated strains of Mycobacterium tuberculosis by human macrophages. *Journal of Immunology*.

[74] Kang PB, Azad AK, Torrelles JB (2005). The human macrophage mannose receptor directs Mycobacterium tuberculosis lipoarabinomannan-mediated phagosome biogenesis. *The Journal of Experimental Medicine*.

[75] Sweet L, Singh PP, Azad AK, Rajaram MVS, Schlesinger LS, Schorey JS (2010). Mannose receptor-dependent delay in phagosome maturation by Mycobacterium avium glycopeptidolipids. *Infection and Immunity*.

[76] Divangahi M, Mostowy S, Coulombe F (2008). NOD2-deficient mice have impaired resistance to Mycobacterium tuberculosis infection through defective innate and adaptive immunity. *Journal of Immunology*.

[77] Ferwerda G, Girardin SE, Kullberg B-J (2005). NOD2 and toll-like receptors are nonredundant recognition systems of Mycobacterium tuberculosis. *PLoS Pathogens*.

[78] Yang Y, Yin C, Pandey A, Abbott D, Sassetti C, Kelliher MA (2007). NOD2 pathway activation by MDP or Mycobacterium tuberculosis infection involves the stable polyubiquitination of Rip2. *Journal of Biological Chemistry*.

[79] Pandey AK, Yang Y, Jiang Z (2009). Nod2, Rip2 and Irf5 play a critical role in the type I interferon response to Mycobacterium tuberculosis. *PLoS Pathogens*.

[80] Shams H, Wizel B, Lakey DL (2003). The CD14 receptor does not mediate entry of Mycobacterium tuberculosis into human mononuclear phagocytes. *FEMS Immunology and Medical Microbiology*.

[81] Khanna M, Srivastava LM (1996). Release of superoxide anion from activated mouse peritoneal macrophages during Mycobacterium tuberculosis infection. *Indian Journal of Experimental Biology*.

[82] Dinadayala P, Lemassu A, Granovski P, Cérantola S, Winter N, Daffé M (2004). Revisiting the structure of the anti-neoplastic glucans of Mycobacterium bovis Bacille Calmette-Guérin: structural analysis of the extracellular and boiling water extract-derived glucans of the vaccine substrains. *Journal of Biological Chemistry*.

[83] van de Veerdonk FL, Teirlinck AC, Kleinnijenhuis J (2010). Mycobacterium tuberculosis induces IL-17A responses through TLR4 and dectin-1 and is critically dependent on endogenous IL-1. *Journal of Leukocyte Biology*.

[84] Rothfuchs AG, Bafica A, Feng CG (2007). Dectin-1 interaction with Mycobacterium tuberculosis leads to enhanced IL-12p40 production by splenic dendritic cells. *Journal of Immunology*.

[85] Yadav M, Schorey JS (2006). The *β*-glucan receptor dectin-1 functions together with TLR2 to mediate macrophage activation by mycobacteria. *Blood*.

[86] Marakalala MJ, Guler R, Matika L (2011). The Syk/CARD9-coupled receptor Dectin-1 is not required for host resistance to Mycobacterium tuberculosis in mice. *Microbes and Infection*.

[87] Dorhoi A, Desel C, Yeremeev V (2010). The adaptor molecule CARD9 is essential for tuberculosis control. *The Journal of Experimental Medicine*.

[88] Marakalala MJ, Graham LM, Brown GD (2010). The role of Syk/CARD9-coupled C-type lectin receptors in immunity to mycobacterium tuberculosis infections. *Clinical and Developmental Immunology*.

[89] Zimmerli S, Edwards S, Ernst JD (1996). Selective receptor blockade during phagocytosis does not alter the survival and growth of Mycobacterium tuberculosis in human macrophages. *American Journal of Respiratory Cell and Molecular Biology*.

[90] Armstrong JA, D’Arcy Hart P (1975). Phagosome lysosome interactions in cultured macrophages infected with virulent tubercle bacilli. Reversal of the usual nonfusion pattern and observations on bacterial survival. *The Journal of Experimental Medicine*.

[91] Geijtenbeek TBH, Torensma R, van Vliet SJ (2000). Identification of DC-SIGN, a novel dendritic cell-specific ICAM-3 receptor that supports primary immune responses. *Cell*.

[92] Geijtenbeek TBH, Krooshoop DJEB, Bleijs DA (2000). DC-SIGN-1CAM-2 interaction mediates dendritic cell trafficking. *Nature Immunology*.

[93] Geijtenbeek T, van Vliet SJ, Koppel EA (2003). Mycobacteria target DC-SIGN to suppress dendritic cell function. *The Journal of Experimental Medicine*.

[94] Tanne A, Ma B, Boudou F (2009). A murine DC-SIGN homologue contributes to early host defense against Mycobacterium tuberculosis. *The Journal of Experimental Medicine*.

[95] Tanne A, Neyrolles O (2010). C-type lectins in immune defense against pathogens the murine DC-SIGN homologue SIGNR3 confers early protection against mycobacterium tuberculosis infection. *Virulence*.

[96] Barreiro LB, Neyrolles O, Babb CL (2006). Promoter variation in the DC-SIGN-encoding gene CD209 is associated with tuberculosis. *PLoS Medicine*.

[97] Barreiro LB, Quach H, Krahenbuhl J (2006). DC-SIGN interacts with mycobacterium ieprae but sequence variation in this lectin is not associated with leprosy in the Pakistani population. *Human Immunology*.

[98] Barreiro LB, Neyrolles O, Babb CL (2007). Length variation of DC-SIGN and L-SIGN neck-region has no impact on tuberculosis susceptibility. *Human Immunology*.

[99] Gaynor CD, McCormack FX, Voelker DR, McGowan SE, Schlesinger LS (1995). Pulmonary surfactant protein A mediates enhanced phagocytosis of Mycobacterium tuberculosis by a direct interaction with human macrophages. *Journal of Immunology*.

[100] Downing JF, Pasula R, Wright JR, Twigg HL, Martin WJ (1995). Surfactant protein A promotes attachment of Mycobacterium tuberculosis to alveolar macrophages during infection with human immunodeficiency virus. *Proceedings of the National Academy of Sciences of the United States of America*.

[101] Pasula R, Wright JR, Kachel DL, Martin WJ (1999). Surfactant protein A suppresses reactive nitrogen intermediates by alveolar macrophages in response to Mycobacterium tuberculosis. *The Journal of Clinical Investigation*.

[102] Stenger S (2005). Immunological control of tuberculosis: role of tumour necrosis factor and more. *Annals of the Rheumatic Diseases*.

[103] Mohan VP, Scanga CA, Yu K (2001). Effects of tumor necrosis factor alpha on host immune response in chronic persistent tuberculosis: possible role for limiting pathology. *Infection and Immunity*.

[104] Casarini M, Ameglio F, Alemanno L (1999). Cytokine levels correlate with a radiologic score in active pulmonary tuberculosis. *American Journal of Respiratory and Critical Care Medicine*.

[105] Kaneko H, Yamada H, Mizuno S (1999). Role of tumor necrosis factor-*α* in Mycobacterium-induced granuloma formation in tumor necrosis factor-*α*-deficient mice. *Laboratory Investigation*.

[106] Senaldi G, Yin S, Shaklee CL, Piguet P-F, Mak TW, Ulich TR (1996). Corynebacterium parvum- and mycobacterium bovis bacillus calmette-Guérin-induced granuloma formation is inhibited in TNF receptor I (TNF-RI) knockout mice and by treatment with soluble TNF-RI. *Journal of Immunology*.

[107] Kindler V, Sappino A-P, Grau GE, Piguet P-F, Vassalli P (1989). The inducing role of tumor necrosis factor in the development of bactericidal granulomas during BCG infection. *Cell*.

[108] Flynn JL, Goldstein MM, Chan J (1995). Tumor necrosis factor-*α* is required in the protective immune response against mycobacterium tuberculosis in mice. *Immunity*.

[109] Chan J, Xing Y, Magliozzo RS, Bloom BR (1992). Killing of virulent Mycobacterium tuberculosis by reactive nitrogen intermediates produced by activated murine macrophages. *The Journal of Experimental Medicine*.

[110] Keane J, Balcewicz-Sablinska MK, Remold HG (1997). Infection by Mycobacterium tuberculosis promotes human alveolar macrophage apoptosis. *Infection and Immunity*.

[111] Saunders BM, Britton WJ (2007). Life and death in the granuloma: immunopathology of tuberculosis. *Immunology and Cell Biology*.

[112] Clay H, Volkman HE, Ramakrishnan L (2008). Tumor necrosis factor signaling mediates resistance to mycobacteria by inhibiting bacterial growth and macrophage death. *Immunity*.

[113] Gardam MA, Keystone EC, Menzies R (2003). Anti-tumour necrosis factor agents and tuberculosis risk: mechanisms of action and clinical management. *Lancet Infectious Diseases*.

[114] van Crevel R, van der Ven-Jongekrijg J, Netea MG, de Lange W, Kullberg B-J, van der Meer JWM (1999). Disease, specific ex vivo stimulation of whole blood for cytokine production: applications in the study of tuberculosis. *Journal of Immunological Methods*.

[115] Flesch I, Kaufmann SHE (1987). Mycobacterial growth inhibition by interferon-*γ*-activated bone marrow macrophages and differential susceptibility among strains of Mycobacterium tuberculosis. *Journal of Immunology*.

[116] Cooper AM, Dalton DK, Stewart TA, Griffin JP, Russell DG, Orme IM (1993). Disseminated tuberculosis in interferon *γ* gene-disrupted mice. *The Journal of Experimental Medicine*.

[117] Jouanguy E, Altare F, Lamhamedi S (1996). Interferon-*γ*-receptor deficiency in an infant with fatal bacille Calmette-Guérin infection. *The New England Journal of Medicine*.

[118] Zhang M, Lin Y, Iyer DV, Gong J, Abrams JS, Barnes PF (1995). T-cell cytokine responses in human infection with Mycobacterium tuberculosis. *Infection and Immunity*.

[119] Ting L-M, Kim AC, Cattamanchi A, Ernst JD (1999). Mycobacterium tuberculosis inhibits IFN-*γ* transcriptional responses without inhibiting activation of STAT1. *Journal of Immunology*.

[120] Munder M, Mallo M, Eichmann K, Modolell M (2001). Direct stimulation of macrophages by IL-12 and IL-18—a bridge built on solid ground. *Immunology Letters*.

[121] Munder M, Mallo M, Eichmann K, Modolell M (1998). Murine macrophages secrete interferon *γ* upon combined stimulation with interleukin (IL)-12 and IL-18: a novel pathway of autocrine macrophage activation. *The Journal of Experimental Medicine*.

[122] Salazar-Mather TP, Hamilton TA, Biron CA (2000). A chemokine-to-cytokine-to-chemokine cascade critical in antiviral defense. *The Journal of Clinical Investigation*.

[123] Pien GC, Satoskar AR, Takeda K, Akira S, Biron CA (2000). Cutting edge: selective IL-18 requirements for induction of compartmental IFN-*γ* responses during viral infection. *Journal of Immunology*.

[124] Ladel CH, Szalay G, Riedel D, Kaufmann SHE (1997). Interleukin-12 secretion by Mycobacterium tuberculosis-infected macrophages. *Infection and Immunity*.

[125] Flynn JL, Goldstein MM, Triebold KJ, Sypek J, Wolf S, Bloom BR (1995). IL-12 increases resistance of BALB/c mice to Mycobacterium tuberculosis infection. *Journal of Immunology*.

[126] Zhang M, Gately MK, Wang E (1994). Interleukin 12 at the site of disease in tuberculosis. *The Journal of Clinical Investigation*.

[127] Bergeron A, Bonay M, Kambouchner M (1997). Cytokine patterns in tuberculous and sarcoid granulomas: correlations with histopathologic features of the granulomatous response. *Journal of Immunology*.

[128] Zhang M, Gong J, Presky DH, Xue W, Barnes PF (1999). Expression of the IL-12 receptor *β*1 and *β*2 subunits in human tuberculosis. *Journal of Immunology*.

[129] Altare F, Ensser A, Breiman A (2001). Interleukin-12 receptor *β*1 deficiency in a patient with abdominal tuberculosis. *Journal of Infectious Diseases*.

[130] Altare F, Lammas D, Revy P (1998). Inherited interleukin 12 deficiency in a child with bacille Calmette- Guerin and Salmonella enteritidis disseminated infection. *The Journal of Clinical Investigation*.

[131] Altare F, Durandy A, Lammas D (1998). Impairment of mycobacterial immunity in human interleukin-12 receptor deficiency. *Science*.

[132] Cooper AM, Magram J, Ferrante J, Orme IM (1997). Interleukin 12 (IL-12) is crucial to the development of protective immunity in mice intravenously infected with mycobacterium tuberculosis. *The Journal of Experimental Medicine*.

[133] Netea MG, Kullberg BJ, Verschueren I, van der Meer JW (2000). Interleukin-18 induces production of proinflammatory cytokines in mice: no intermediate role for the cytokines of the tumor necrosis factor family and interleukin-1beta. *European Journal of Immunology*.

[134] Sugawara I, Yamada H, Kaneko H, Mizuno S, Takeda K, Akira S (1999). Role of interleukin-18 (IL-18) in mycobacterial infection in IL-18- gene-disrupted mice. *Infection and Immunity*.

[135] Kobayashi K, Kai M, Gidoh M-I (1998). The possible role of interleukin (IL)-12 and interferon-*γ*-inducing factor/IL-18 in protection against experimental Mycobacterium leprae infection in mice. *Clinical Immunology and Immunopathology*.

[136] Vankayalapati R, Wizel B, Weis SE, Samten B, Girard WM, Barnes PF (2000). Production of interleukin-18 in human tuberculosis. *Journal of Infectious Diseases*.

[137] Law K, Weiden M, Harkin T, Tchou-Wong K, Chi C, Rom WN (1996). Increased release of interleukin-1*β*, interleukin-6, and tumor necrosis factor-*α* by bronchoalveolar cells lavaged from involved sites in pulmonary tuberculosis. *American Journal of Respiratory and Critical Care Medicine*.

[138] Juffermans NP, Florquin S, Camoglio L (2000). Interleukin-1 signaling is essential for host defense during murine pulmonary tuberculosis. *Journal of Infectious Diseases*.

[139] Yamada H, Mizumo S, Horai R, Iwakura Y, Sugawara I (2000). Protective role of interleukin-1 in mycobacterial infection in IL-1 *α*/*β* double-knockout mice. *Laboratory Investigation*.

[140] Sugawara I, Yamada H, Hua S, Mizuno S (2001). Role of interleukin (IL)-1 type 1 receptor in mycobacterial infection. *Microbiology and Immunology*.

[141] Fremond CM, Yeremeev V, Nicolle DM, Jacobs M, Quesniaux VF, Ryffel B (2004). Fatal Mycobacterium tuberculosis infection despite adaptive immune response in the absence of MyD88. *The Journal of Clinical Investigation*.

[142] Scanga CA, Bafica A, Feng CG, Cheever AW, Hieny S, Sher A (2004). MyD88-deficient mice display a profound loss in resistance to Mycobacterium tuberculosis associated with partially impaired Th1 cytokine and nitric oxide synthase 2 expression. *Infection and Immunity*.

[143] Hölscher C, Reiling N, Schaible UE (2008). Containment of aerogenic Mycobacterium tuberculosis infection in mice does not require MyD88 adaptor function for TLR2, -4 and -9. *European Journal of Immunology*.

[144] Mayer-Barber KD, Barber DL, Shenderov K (2010). Cutting edge: caspase-1 independent IL-1*β* production is critical for host resistance to Mycobacterium tuberculosis and does not require TLR signaling in vivo. *Journal of Immunology*.

[145] Fremond CM, Togbe D, Doz E (2007). IL-1 receptor-mediated signal is an essential component of MyD88-dependent innate response to Mycobacterium tuberculosis infection. *Journal of Immunology*.

[146] Saunders BM, Frank AA, Orme IM, Cooper AM (2000). Interleukin-6 induces early gamma interferon production in the infected lung but is not required for generation of specific immunity to Mycobacterium tuberculosis infection. *Infection and Immunity*.

[147] Schindler R, Mancilla J, Endres S, Ghorbani R, Clark SC, Dinarello CA (1990). Correlations and interactions in the production of interleukin-6 (IL-6), IL-1, and tumor necrosis factor (TNF) in human blood mononuclear cells: IL-6 suppresses IL-1 and TNF. *Blood*.

[148] Wiekowski MT, Leach MW, Evans EW (2001). Ubiquitous transgenic expression of the IL-23 subunit p19 induces multiorgan inflammation, runting, infertility, and premature death. *Journal of Immunology*.

[149] Cooper AM, Roberts AD, Rhoades ER, Callahan JE, Getzy DM, Orme IM (1995). The role of interleukin-12 in acquired immunity to Mycobacterium tuberculosis infection. *Immunology*.

[150] Cooper AM, Kipnis A, Turner J, Magram J, Ferrante J, Orme IM (2002). Mice lacking bioactive IL-12 can generate protective, antigen-specific cellular responses to mycobacterial infection only if the IL-12 p40 subunit is present. *Journal of Immunology*.

[151] Khader SA, Pearl JE, Sakamoto K (2005). IL-23 compensates for the absence of IL-12p70 and is essential for the IL-17 response during tuberculosis but is dispensable for protection and antigen-specific IFN-*γ* responses if IL-12p70 is available. *Journal of Immunology*.

[152] Khader SA, Guglani L, Rangel-Moreno J (2011). IL-23 is required for long-term control of Mycobacterium tuberculosis and B cell follicle formation in the infected lung. *Journal of Immunology*.

[153] Wozniak TM, Ryan AA, Triccas JA, Britton WJ (2006). Plasmid interleukin-23 (IL-23), but not plasmid IL-27, enhances the protective efficacy of a DNA vaccine against Mycobacterium tuberculosis infection. *Infection and Immunity*.

[154] Wozniak TM, Ryan AA, Britton WJ (2006). Interleukin-23 restores immunity to Mycobacterium tuberculosis infection in IL-12p40-deficient mice and is not required for the development of IL-17-secreting T cell responses. *Journal of Immunology*.

[155] Wozniak TM, Saunders BM, Ryan AA, Britton WJ (2010). Mycobacterium bovis BCG-specific Th17 cells confer partial protection against Mycobacterium tuberculosis infection in the absence of gamma interferon. *Infection and Immunity*.

[156] Chen X, Zhang M, Liao M (2010). Reduced Th17 response in patients with tuberculosis correlates with IL-6R expression on CD4^+^ T cells. *American Journal of Respiratory and Critical Care Medicine*.

[157] Zhang M, Zheng X, Zhang J (2012). CD19^+^CD1d^+^CD5^+^ B cell frequencies are increased in patients with tuberculosis and suppress Th17 responses. *Cellular Immunology*.

[158] Powrie F, Coffman RL (1993). Inhibition of cell-mediated immunity by IL4 and IL10. *Research in Immunology*.

[159] Gordon S (2003). Alternative activation of macrophages. *Nature Reviews Immunology*.

[160] Kahnert A, Seiler P, Stein M (2006). Alternative activation deprives macrophages of a coordinated defense program to Mycobacterium tuberculosis. *European Journal of Immunology*.

[161] Essner R, Rhoades K, McBride WH, Morton DL, Economou JS (1989). IL-4 down-regulates IL-1 and TNF gene expression in human monocytes. *Journal of Immunology*.

[162] Joyce DA, Steer JH (1996). IL-4, IL-10 and IFN-*γ* have distinct, but interacting, effects on differentiation-induced changes in TNF-*α* and TNF receptor release by cultured human monocytes. *Cytokine*.

[163] Rook GAW (2007). Th2 cytokines in susceptibility to tuberculosis. *Current Molecular Medicine*.

[164] Surcel H-M, Troye-Blomberg M, Paulie S (1994). Th1/Th2 profiles in tuberculosis, based on the proliferation and cytokine response of blood lymphocytes to mycobacterial antigens. *Immunology*.

[165] Dheda K, Chang J-S, Breen RAM (2005). Expression of a novel cytokine, IL-4delta2, in HIV and HIV-tuberculosis co-infection. *AIDS*.

[166] Mazzarella G, Bianco A, Perna F (2003). T lymphocyte phenotypic profile in lung segments affected by cavitary and non-cavitary tuberculosis. *Clinical and Experimental Immunology*.

[167] Hernández-Pando R, Orozcoe H, Sampieri A (1996). Correlation between the kinetics of Th1/Th2 cells and pathology in a murine model of experimental pulmonary tuberculosis. *Immunology*.

[168] Howard AD, Zwilling BS (1999). Reactivation of tuberculosis is associated with a shift from type 1 to type 2 cytokines. *Clinical and Experimental Immunology*.

[169] van Crevel R, Karyadi E, Preyers F (2000). Increased production of interleukin 4 by CD4^+^ and CD8^+^ T cells from patients with tuberculosis is related to the presence of pulmonary cavities. *Journal of Infectious Diseases*.

[170] Fujiwara N, Kobayashi K (2005). Macrophages in inflammation. *Current Drug Targets*.

[171] Rojas M, Olivier M, Gros P, Barrera LF, García LF (1999). TNF-*α* and IL-10 modulate the induction of apoptosis by virulent Mycobacterium tuberculosis in murine macrophages. *Journal of Immunology*.

[172] Murray PJ, Wang L, Onufryk C, Tepper RI, Young RA (1997). T cell-derived IL-10 antagonizes macrophage function in mycobacterial infection. *Journal of Immunology*.

[173] Cyktor JC, Carruthers B, Beamer GL, Turner J (2013). Clonal expansions of CD8^+^ T cells with IL-10 secreting capacity occur during chronic Mycobacterium tuberculosis infection. *PloS One*.

[174] O’Leary S, O’Sullivan MP, Keane J (2011). IL-10 blocks phagosome maturation in Mycobacterium tuberculosis-infected human macrophages. *American Journal of Respiratory Cell and Molecular Biology*.

[175] Gazzinelli RT, Oswald IP, James SL, Sher A (1992). IL-10 inhibits parasite killing and nitrogen oxide production by IFN-*γ*- activated macrophages. *Journal of Immunology*.

[176] Redpath S, Ghazal P, Gascoigne NRJ (2001). Hijacking and exploitation of IL-10 by intracellular pathogens. *Trends in Microbiology*.

[177] Moore KW, de Waal Malefyt R, Coffman RL, O’Garra A (2001). Interleukin-10 and the interleukin-10 receptor. *Annual Review of Immunology*.

[178] Khader SA, Partida-Sanchez S, Bell G (2006). Interleukin 12p40 is required for dendritic cell migration and T cell priming after Mycobacterium tuberculosis infection. *The Journal of Experimental Medicine*.

[179] Redford PS, Boonstra A, Read S (2010). Enhanced protection to Mycobacterium tuberculosis infection in IL-10-deficient mice is accompanied by early and enhanced Th1 responses in the lung. *European Journal of Immunology*.

[180] Zhang M, Gong J, Iyer DV, Jones BE, Modlin RL, Barnes PF (1994). T cell cytokine responses in persons with tuberculosis and human immunodeficiency virus infection. *The Journal of Clinical Investigation*.

[181] Bonecini-Almeida MG, Ho JL, Boéchat N (2004). Down-modulation of lung immune responses by interleukin-10 and transforming growth factor *β* (TGF-*β*) and analysis of TGF-*β* receptors I and II in active tuberculosis. *Infection and Immunity*.

[182] Huard RC, Chitale S, Leung M (2003). The Mycobacterium tuberculosis complex-restricted gene cfp32 encodes an expressed protein that is detectable in tuberculosis patients and is positively correlated with pulmonary interleukin-10. *Infection and Immunity*.

[183] Turner J, Gonzalez-Juarrero M, Ellis DL (2002). In vivo IL-10 production reactivates chronic pulmonary tuberculosis in C57BL/6 mice. *Journal of Immunology*.

[184] Beamer GL, Flaherty DK, Assogba BD (2008). Interleukin-10 promotes Mycobacterium tuberculosis disease progression in CBA/J mice. *Journal of Immunology*.

[185] Roque S, Nobrega C, Appelberg R, Correia-Neves M (2007). IL-10 underlies distinct susceptibility of BALB/c and C57BL/6 mice to Mycobacterium avium infection and influences efficacy of antibiotic therapy. *Journal of Immunology*.

[186] Jacobs M, Fick L, Allie N, Brown N, Ryffel B (2002). Enhanced immune response in Mycobacterium bovis bacille calmette guerin (BCG)-infected IL-10-deficient mice. *Clinical Chemistry and Laboratory Medicine*.

[187] North RJ (1998). Mice incapable of making IL-4 or IL-10 display normal resistance to infection with Mycobacterium tuberculosis. *Clinical and Experimental Immunology*.

[188] Higgins DM, Sanchez-Campillo J, Rosas-Taraco AG, Lee EJ, Orme IM, Gonzalez-Juarrero M (2009). Lack of IL-10 alters inflammatory and immune responses during pulmonary Mycobacterium tuberculosis infection. *Tuberculosis*.

[189] Toossi Z, Gogate P, Shiratsuchi H, Young T, Ellner JJ (1995). Enhanced production of TGF-*β* by blood monocytes from patients with active tuberculosis and presence of TGF-*β* in tuberculous granulomatous lung lesions. *Journal of Immunology*.

[190] Dahl KE, Shiratsuchi H, Hamilton BD, Ellner JJ, Toossi Z (1996). Selective induction of transforming growth factor *β* in human monocytes by lipoarabinomannan of Mycobacterium tuberculosis. *Infection and Immunity*.

[191] Ding A, Nathan CF, Graycar J, Derynck R, Stuehr DJ, Srimal S (1990). Macrophage deactivating factor and transforming growth factors-*β*1, -*β*2, and -*β*3 inhibit induction of macrophage nitrogen oxide synthesis by IFN-*γ*. *Journal of Immunology*.

[192] Rojas RE, Balaji KN, Subramanian A, Boom WH (1999). Regulation of human CD4^+^
*αβ* T-cell-receptor-positive (TCR^+^) and *γδ* TCR^+^ T-cell responses to Mycobacterium tuberculosis by interleukin-10 and transforming growth factor *β*. *Infection and Immunity*.

[193] Ruscetti F, Varesio L, Ochoa A, Ortaldo J (1993). Pleiotropic effects of transforming growth factor-*β* on cells of the immune system. *Annals of the New York Academy of Sciences*.

[194] Hirsch CS, Ellner JJ, Blinkhorn R, Toossi Z (1997). In vitro restoration of T cell responses in tuberculosis and augmentation of monocyte effector function against Mycobacterium tuberculosis by natural inhibitors of transforming growth factor *β*. *Proceedings of the National Academy of Sciences of the United States of America*.

[195] Desvignes L, Wolf AJ, Ernst JD (2012). Dynamic roles of type I and type II IFNs in early infection with Mycobacterium tuberculosis. *Journal of Immunology*.

[196] Stern JNH, Keskin DB, Romero V (2009). Molecular signatures distinguishing active from latent tuberculosis in peripheral blood mononuclear cells, after in vitro antigenic stimulation with purified protein derivative of tuberculin (PPD) or Candida: a preliminary report. *Immunologic Research*.

[197] Berry MPR, Graham CM, McNab FW (2010). An interferon-inducible neutrophil-driven blood transcriptional signature in human tuberculosis. *Nature*.

[198] McNab FW, Ewbank J, Rajsbaum R (2013). TPL-2-ERK1/2 signaling promotes host resistance against intracellular bacterial infection by negative regulation of type I IFN production. *Journal of Immunology*.

[199] Giosuè S, Casarini M, Alemanno L (1998). Effects of aerosolized interferon-*α* in patients with pulmonary tuberculosis. *American Journal of Respiratory and Critical Care Medicine*.

[200] Palmero DJ, Eiguchi K, Rendo P, Castro Zorrilla L, Abbate E, González Montaner LJ (1999). Phase II trial of recombinant interferon-*α*2b in patients with advanced intractable multidrug-resistant pulmonary tuberculosis: long-term follow-up. *International Journal of Tuberculosis and Lung Disease*.

[201] Ward CM, Jyonouchi H, Kotenko SV (2007). Adjunctive treatment of disseminated Mycobacterium avium complex infection with interferon alpha-2b in a patient with complete interferon-gamma receptor R1 deficiency. *European Journal of Pediatrics*.

[202] Telesca C, Angelico M, Piccolo P (2007). Interferon-alpha treatment of hepatitis D induces tuberculosis exacerbation in an immigrant. *Journal of Infection*.

[203] Redford PS, Mayer-Barber KD, McNab FW (2013). Influenza A virus impairs control of Mycobacterium tuberculosis co-infection through a type I interferon receptor dependent pathway. *The Journal of Infectious Diseases*.

[204] Bouchonnet F, Boechat N, Bonay M, Hance AJ (2002). Alpha/beta interferon impairs the ability of human macrophages to control growth of Mycobacterium bovis BCG. *Infection and Immunity*.

[205] Giacomini E, Remoli ME, Gafa V, Pardini M, Fattorini L, Coccia EM (2009). IFN-*β* improves BCG immunogenicity by acting on DC maturation. *Journal of Leukocyte Biology*.

[206] Novikov A, Cardone M, Thompson R (2011). Mycobacterium tuberculosis triggers host type I IFN signaling to regulate IL-1*β* production in human macrophages. *Journal of Immunology*.

[207] Mayer-Barber KD, Andrade BB, Barber DL (2011). Innate and adaptive interferons suppress IL-1*α* and IL-1*β* production by distinct pulmonary myeloid subsets during Mycobacterium tuberculosis infection. *Immunity*.

[208] Denis M (1991). Recombinant murine beta interferon enhances resistance of mice to systemic Mycobacterium avium infection. *Infection and Immunity*.

[209] Manca C, Tsenova L, Bergtold A (2001). Virulence of a Mycobacterium tuberculosis clinical isolate in mice is determined by failure to induce Th1 type immunity and is associated with induction of IFN-*α*/*β*. *Proceedings of the National Academy of Sciences of the United States of America*.

[210] Kuchtey J, Fulton SA, Reba SM, Harding CV, Boom WH (2006). Interferon-*αβ* mediates partial control of early pulmonary Mycobacterium bovis bacillus Calmette-Guérin infection. *Immunology*.

[211] Ordway D, Henao-Tamayo M, Harton M (2007). The hypervirulent Mycobacterium tuberculosis strain HN878 induces a potent TH1 response followed by rapid down-regulation. *Journal of Immunology*.

[212] Guarda G, Braun M, Staehli F (2011). Type I interferon inhibits interleukin-1 production and inflammasome activation. *Immunity*.

[213] Ho HH, Ivashkiv LB (2006). Role of STAT3 in type I interferon responses: negative regulation of STAT1-dependent inflammatory gene activation. *Journal of Biological Chemistry*.

